# Ethical Implications in the Use of Embryonic and Adult Neural Stem Cells

**DOI:** 10.1155/2012/470949

**Published:** 2012-09-10

**Authors:** Rodrigo Ramos-Zúñiga, Oscar González-Pérez, Ana Macías-Ornelas, Vivian Capilla-González, Alfredo Quiñones-Hinojosa

**Affiliations:** ^1^Department of Neurosciences, CUCS, Universidad de Guadalajara, 44630 Guadalajara, JAL, Mexico; ^2^Department of Neurosurgery, Brain Tumor Stem Cells Laboratory, Johns Hopkins University, Baltimore, MD 4940, USA

## Abstract

The advent and growth of technological advances have led to new routes of knowledge. Thereby, we currently face new challenges. We have just started to get a glimpse of the structural and functional role of neural stem cells in differentiation and migration processes, the origin of synaptic networks, and subsequent readjustments in specific circuits. A whole range of treatment possibilities originates from this knowledge that potentially can be used for different neurological diseases in humans. Although this is an encouraging scenario, it implies that the human brain is the object of such study, as well as its potential manipulation and transplantation. It is, therefore, pertinent that ethical principles should be followed in such research to have proper balance between what can be done and what should be done, according to every specific context. Hence, it is wise to consider ethical implications in every research project, along with potential clinical applications, under the principle of causing no harm, following risk and benefit rules in decision making and with respect of the human condition as a priority.

## 1. Stem Cells

Biotechnology and gene manipulation, as a result of elucidating the human genome, have set new challenges in view of the enormous amount of information in neurosciences area. On one hand, this new knowledge from the last ten years is huge in terms of molecular and genetic information that led to the identification of stem cells. This happened after Thomson [1, 2] proposed the scientific analysis of embryos not used in protocols for *in vitro* fertilization (IVF). Several cell lines were isolated, compatible with all the different cell phenotypes of the adult. At the time, three features were identified for embryonic stem cells. (a) They derive from embryos in the preimplantation stage. (b) They are preserved as undifferentiated cells for indefinite time in special media. (c) They maintain their pluripotential capacity to generate any line of cells from all embryonic germinal layers [3].

The knowledge around stem cells and their differentiation and maturation processes to correctly make structured tissues is the result of many years of phylogenetic evolution and significant knowledge of the human ontogeny. Currently, we are familiar with many of the variants of pluripotential cells in different types of tissue, as well as advances and different applications of such cells, many of them still under investigation. The greater understanding of the human genome and the normal phenotype evolution gave way to the identification of abnormal phenotype expression in early stages of neurodevelopment disturbances, and also programmed or late stages such as in the case of many degenerative diseases [4–6]. 

The identification of neural stem cells had a breakthrough associated with studies of animal embryos and subsequently in human beings, and the anatomic and functional confirmation of neuro-ontogeny. When this information was learned, the first debate came about: where do we get tissue from? What is the origin and destination of such tissue? 

In a first phase, investigators resorted to embryo studies from miscarriages, but the changes in IVF strategies for the benefit of couples with fertility problems led to the availability of embryos in association with the gestational process. The question about the origin of such tissue was still unanswered and new questions arose about the care and management of embryos and the final destination of those not selected or successfully implanted, like the ones preserved in the laboratory. This gave way to begin having certain availability of these embryos for scientific research, with variable legal and ethical implications. There are countries that forbid this practice altogether, under the premise that you cannot make decisions about life to propose the cure of certain diseases. In this scenario, the debate about the beginning of life comes up again, as well as the limitations to use embryos in a totally limitless way or the opposite position in countries where embryos are allowed to be created *ex profeso *for research purposes along with the procurement of stem cells [7].

So far, regulations have been issued as legal standards trying to approach the way in which these processes can follow the same line of respect for human integrity, without limiting the potentialities of science and therapy in embryo studies [8]. 

Recently, the American Association of Cancer Research (AACR) has promoted new agreements endorsed by the U.S. government to validate the continuity of studies in embryonic stem cells, as a key element to find treatment for nearly 200 diseases linked to the concept of “cancer.” The rationale is that the origin of cancer follows genetic and epigenetic factors which must be studied in order to find a cure for this disease. Consequently, knowing the differences between normal stem cells and cancer stem cells will allow to provide greater therapeutic options in the future. The agreement postulates the prevalence of respect for the human embryo, abiding to the international standard of making use of embryos up to day 14 of the blastocyst stage, but once again emphasizing the pertinence of continuous research of scientific information that cannot be documented otherwise (AACR, 2010) [9]. 

Aside from the knowledge of embryonic stem cells, other scientific contributions began changing the paradigm in the information and exploration of other exciting scientific territories. The theoretical concept that the adult central nervous system never regenerates [10] changed when scientists finding the neurogenic capacity of the adult brain. Even in human, neural stem cells were found primarily in two region, the subventricular zone of the lateral ventricles and the dentate gyrus of the hippocampus [11–14]. In the case of rodents, newly generated cells are able to migrate, differentiate, mature, and integrate into preexisting circuits, where supply functions as cognition and olfaction [15]. 

## 2. Adult Stem Cells and Transplants

It is relevant to analyze the role of neural stem cells because of their potential clinical application in regenerative and repair therapy. It is clear that it is important to have discussions with the purpose of avoiding ethical dilemmas in science and in society, as well as their consequences concerning the procurement of these cells so that investigators can approach the field of applied science, in this case in reference to transplants. 

Even though at the present time organ and tissue transplants are a real fact in our everyday life, this was accomplished thanks to a series of collaborations and willingness that led to experimental surgical refinements, the development of microsurgery, as well as the training and skill to perform vascular anastomosis and control rejection responses, just to mention some of the most relevant events

Nevertheless, in the case of neural stem cell transplants experience is barely beginning in the sense of systematization of processes, standardization of sources and origin of stem cells, application methods, controlled directionality of effects, clinical impact, and inherent risks and complications of the surgical procedures [16–20]. 

Part of this promising expectation was originally based on the concept that the brain was a site with a peculiar structure and functionality which made it “privileged,” thus an organ with certain good features for receiving transplants. All of this was the result of having structures such as the blood-brain barrier and unique conditions in the behavior and tolerance of immune mechanisms [21].

However, these concepts have changed because we now have more information about the blood-brain barrier not being so airtight, and that it allows the passage of certain type of mediator cells of the immune response, and that it changes when there is an intrinsic inflammatory process [22]. The immune response concept itself underwent a change from learning that the microglia are capable of presenting antigens and activating phagocytic features and inducing *in situ* cytokine activities [23]. Moreover, the brain is currently no longer considered as a “privileged” site but an organ with different immune mechanisms and very peculiar immune response processes. In addition, it has been observed that many pathological and inflammatory conditions significantly affect neurogenic niches. Even more, increasing evidences indicate that chemokines and cytokines play an important role in regulating proliferation, cell fate choices, migration, and survival of neural stem cells under physiological conditions [24].

Even so, the brain is still a site with more favorable conditions for transplantation, compared to other peripheral organs, according to the tolerance experience found in fetal tissues with moderate immune suppression therapy, unlike xenografs, which are rejected. This opened perspectives for transplantation of neural stem cells, presumed to have greater potential in terms of structural and functional regeneration, with reduced risk of immune rejection reaction [25–27]. 

According to the concepts above, it has been considered that the potential action of these cells, when transplanted to the human nervous system, would have three relevant effects.

**(I) Cell Regeneration**

Previous studies showed that neural stem cell can differentiate and migrate after transplantation to integrate into the host tissue in models of spinal lesions and stroke [28–32]. This supports and gives hope to the proposed repair therapy. Additionally, active electric connection mechanisms in the brain cortex have been identified [33], but the functional relevance of this response is still to be established, as well as the reason for the persistence of certain in situ cells as undifferentiated [34].

**(II) Neuroprotection**

An additional function of neural stem cells is the neuroprotective properties. Transplanted neural stem cells can release specific factors, which promote the survival and prevent cell death in sites where they have been implanted. Some of these released factors that increase their bioavailability are neurotrophic factors [35]. This proposal has led to expectations in case of inflammatory disorder [36], neonatal brain injury [37], and degenerative diseases [38–40]. One of the mechanisms proposed is that the transplanted neurospheres-derived cells have a two way molecular exchange that makes them more sensitive for releasing neurotrophic factors when found in the neuro-glial microenvironment.

**(III) Immunomodulation**

There is growing evidence about the immune modulating capacity of neural stem cells from in vitro and in vivo studies, in terms of regulating the deleterious inflammatory response and fostering immune conditions for tissue regeneration [41]. This has been shown as a reduced inflammatory response in experimental autoimmune encephalomyelitis, and a reduced proliferation of T-cell derivatives in response to concanavalin A in the oligodendrocytes [42]. Likewise, a reduced inflammatory response has been identified in experimental spinal lesions [43, 44], which would open the way to a new modality in the mechanism of action of transplanted neural stem cells.

## 3. Potential Applications of Adult Neural Stem Cells

At one point, it was considered that embryonic stem cells were the key for the creation of different potential cell lines with therapeutic purposes, when it became evident that they were available in greater number, easy to identify, and grow in culture. Also, embryonic stem cells grew faster and more easily, compared to adult stem cells, and ultimately they could be more plastic and manageable. 

Even so, the use of embryonic stem cells presents legal and ethical limitations, such as the obvious destruction of live embryos to obtain stem cells. Additionally, other technical limitations not previously seen were identified: (a) rejection of embryonic stem cells requiring immunosuppressive treatment, (b) the possibility to induce cells of tumor lineage. All these limitations lead investigators to look for other alternatives. 

The search for adult stem cells has become all the more important, getting greater availability to their source of origin and limiting ethical conflicts with the use of embryos [45]. 

Adult stem cells are found in the brain, pancreas, liver, bone marrow, blood, muscle, skin, and other body tissue. They can be crucial to continue forming and generating tissue which is structurally linked to cell lineages from where they have been phenotypically collected. 

Currently, a few scenarios are found in which adult stem cells can have potential application leading to the identification and characterization of adult tissue with germinal properties such as the case of the hematopoietic tissue and the skin. Olfactory tissue stem cells have also been proposed as an alternative to be studied and transplanted to repair vascular brain lesions or traumatic spinal cord lesions [30]. A unique feature of adult neural stem cells is that they have been well identified and characterized in the adult brain, particularly in the subventricular zone and the dentate gyrus of the hippocampus [15], where neural stem cells show a potential differentiation to glial and neuronal cells aside from being compatible with radial migration [46, 47]. 

This sort of application has the potential advantage of implanting predifferentiated cells to certain glial or neuronal cell lineage, as a step forward in the therapy to repair pathologic processes, for example, ischemia, multiple sclerosis, spinal cord injury, or degenerative processes such as Alzheimer, Huntington, or Parkinson's disease [48–51]. 

## 4. Risks with Ethical Implications

Risks involved in the clinical application of adult neural stem cells have not been totally evaluated, nor have long-term followup, so it is still necessary to be cautious and alert. Not only because of the ethical implications that can be anticipated in terms to what is morally and socially accepted in each community, but also because of the technical implications and risks to the patient's health. 

These risks are basically found within the following possibilities:

**(A) Risk of Tumors**

This possibility has been considered a real one, according to reports of teratomas in the striate cortex in experimental Parkinson models. A previous report mentioned 20% of new onset tumors in an experimental sample, when undifferentiated stem cells were used [52]. The possibility of using viral vectors or genetic manipulations from regulator genes, trying to guide differentiation and efficacy in dopaminergic neurons, also involves the risk of viral transmission out of control and out of target, plus the risk of mutagenesis. The stem cells themselves pose an additional unknown risk. The longer cells are grown in culture, the more likely they are to acquire genetic and epigenetic changes, in agreement with the previous experience with embryonic stem cells [53–55].

**(B) Inadequate Migration**

The risk of migration defects gives rise to heterotopias in the white matter, sub-ependymal region, and the cortical gray substance if there is no control in the migration process toward a lesion in a specific area. There might be an out of target aberrant migration, giving rise to potential heterotopias with the ensuing clinical complications such as difficult to control epilepsy (refractory) or other neuropathological conditions. Being able to get greater differentiation in adult neural stem cells and the necessary refinement in target migration is still a challenge [27, 56].

**(C) Transplant Rejection**

Immune rejection conditions will always be found in adult neural stem cell transplants. Although there is now greater experience with embryonic mesencephalic stem cells and management of the need to give constant immunosuppression (cyclosporine), not only to avoid rejection, but also to maintain clinical response in adult neural stem cells the experience is not the same. Theoretically, since cells are more differentiated in adult tissues and more antigenic they might require greater use of immunosuppressive drugs with the inherent additional risks such as liver and renal toxicity, hypertension and immunodeficiency [57].

**(D) Surgical Risks**

In spite that most of the brain cell tissue transplants are done with stereotactic method, with specific mapping and accurate coordinates, the procedure is not devoid of risk. An average of 3% surgical risk has been reported associated to bleedings or infection. Even though the risk involved is less compared to deep brain stimulation procedures, where a foreign body is placed, this condition must be carefully looked into for risk-benefit analysis [34].

**(E) Infections**

This is a constant risk in every cell transplant process in which pathogens may be transmitted from the donor to the recipient, such as hepatitis B or C, lymphotropic virus, HIV/Aids, cytomegalovirus, and herpes simplex virus. In addition, there is also the risk of infection in the culture media and in handling the samples, either from bacteria (Staphylococcus, Streptococci, *E. coli*), yeasts, spores, and prion diseases [58].

## 5. Neuroethics and Neural Stem Cells

In view of the response from society to these unforeseen topics resulting from scientific and technological advances applied to medical science, it was necessary to have ethical support from certain deontological criteria and universal concepts (not specific to medical science), such as the UN Declaration of Universal Human Rights [59]. 

Subsequently, a more specific form is described with the advent of bioethics [60], applied in greater association with life science and survival. The latter school of thought is prevalent mainly in western culture and postulates respect of basic principles of human behavior in interaction with other individuals. This is the reason why every human action, even nonmedical, may be subjected to these precepts. 

In more recent times, neuroethics has come to respond to the great demand of topics that neurosciences have put forth in dealing with traditional ethics. It has been necessary to establish more specific study lines, as a result of a great amount of information, research, and potential treatment applications with ethical implications, but more specific and dealt by experts who have deep knowledge of dilemmas prevailing in basic and clinical neuroscience.

Neuroethics is not proposed with the reductionist view of a single organ, but rather redefining the important role of neuroscience as the object of study in every variant, and the unique condition that it is the human brain itself that ponders, discusses and decides about its own object of study. 

Today, there are two points of interest in the field of stem cells proposed by neuroethics: on the one hand, the origin of stem cells and the way in which they are obtained, studied, protected, and preserved. On the other hand, everything around the application of neural stem cells, from feasibility to viability, risk and benefit, the transplant process itself, complications, outcome, public health impact, and also potential deviations [61–63]. 

At this point, I will particularly refer to the second segment, since there is a great deal of literature about the management and regulation of the origin of embryonic stem cells particularly referring to regulations of human embryos for research, where the dilemma of the difference between zygote, embryo, fetus, and the moment when life starts is still debated. However, in terms of ethical dilemmas from the application of stem cells to neurologic diseases, we do not have the same amount of information (Table 1).

At the present time, it is relevant to promote and motivate the use of adult neural stem cells for three main reasons: (a) to reorient the strategy to a more successful outcome considering cell lineage in the most differentiated possible way, in order to have better certainty about the functional implications of mature cells in the recipient tissue; (b) when the same tissue origin is considered, there would be greater likelihood to reach the therapeutic target, with less risk of variables involved in migration and functional errors, once the rejection reaction is under control; (c) adult neural stem cells involve less ethical objections, as compared to embryonic stem cells [64, 65]. 

For these specific potential cases for research and transplants, and the challenges and dilemmas they involve, it is wise to once again resort to the pragmatic ethics in bioethical research, that rules its actions within a framework of respect for the subjects of research, particularly groups of sick and vulnerable subjects. 

Thus, it is considered healthy that proposals of fully justified new research that produces scientific knowledge, improves public health as well as the quality of medical care, must, above everything else, protect the patients and avoid creating damage. Also, such research must be totally compatible not only with legal regulations but also with precepts of moral and behavior values and virtues that identify a defined social context [66, 67]. 

Following these concepts, in the case of adult neural stem cell transplants, the same criteria used for transplants in general are applied in which a bioethical triangle is formed. 

On one hand, we find the donor line in which there is total freedom, knowledge, informed consent to give, by means of an altruistic action, the tissue, and cells for a transplant. No extraordinary risks for the life or health of the donor are involved, without conflicts of interest or submission to scientific postures. In every case, a reasonable risk-benefit position must prevail, taking into account potential help for control or cure of a disease and the value of contributions to scientific knowledge. 

On the other hand, we find the recipient line which would be assumed to be the main potential beneficiary, with the same rights, with informed consent and ethically validated in terms of selection and assignment criteria and with clear information about the expectations of the procedure, without any conflict of interest from secondary compensations. The donor makes the decision according to the established riskbenefit, considering that what is technically viable must also be ethically acceptable. 

Finally, at the base of the triangle, we find the line of the human and professional team, participating in the scientific research, the harvesting of cells, cultures, transplant procurement, the transplantation itself, and subsequent followup and control. As any human activity, it is not free of risks and byas. That is the reason why everyone must do their work with self-respect, and respect for the team and obviously everyone involved in the transplantation process; following the principles of professional behavior of technical training, ethically acknowledged and supported. 

This rational balance in the bioethical triangle must be totally compatible with principles of truthfulness, justice, equality, autonomy, welfare, and confidentiality, postulated by bioethics and that now neuroethics adopts as fundamental premises to deal with these new dilemmas that science has brought about neural stem cells [68]. 

**Figure 1:**
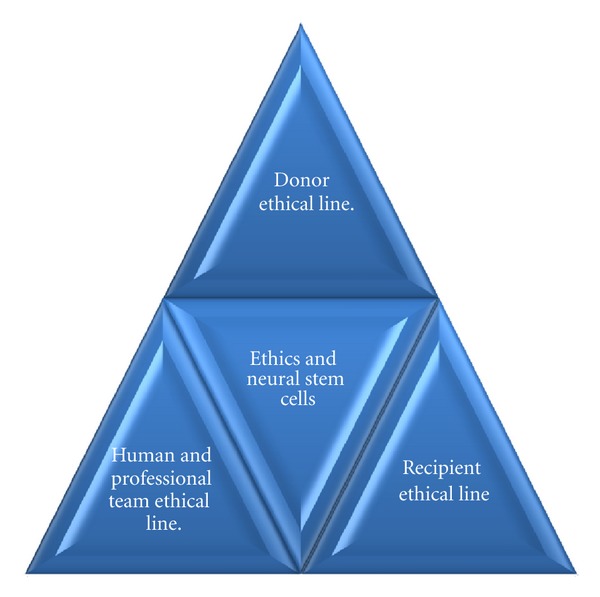
Bioethical triangle in the research and use of neural stem cells.

This is where neuroethics attempts to create awareness among neuroscientists, geneticists, neurosurgeons, and all the professionals involved in this process about the responsibility of anticipating to the debate due to the use and abuse of these procedures and research. The neuroethicists have a social responsibility to see that the advent of technological advances which have increased our ability and power to carry out new experiments be maintained within the risk-benefit regulatory criteria, with respect for life and within morally and socially accepted strategies [69] (Figure 1).

One of the current and future challenges that has also been settled with specific actions in this scenario has to do with justice and access to this type of therapy as a standard treatment for given diseases in the future. One of the ways to deal with this matter has been to discourage development and approval of restrictive patents in this area, in order for this knowledge to prevail as heritage of humanity, and that viability and feasibility can be guaranteed everywhere in the world following the simplest reproducibility strategies with an ethical point of view [70]. 

## 6. Conclusions

Extensive and constant discussion is required about the role of biotechnology and its ethical implications in the scenario of neural stem cells. If we can have a balanced position, we shall be able to do scientific work with a tool that has great potential to solve health problems in different stages of life, such as in the case of neural stem cells, with no limitations. But it should certainly prevail in a position of respect for human beings and society that are the main beneficiaries of this promising proposal.

## Figures and Tables

**Table 1:** Ethical considerations in the use of neural stem cells. Ethical considerations related to the origin and source of neural stem cells and the potential clinical applications.

Ethical considerations	
Origin	Embryos from miscarriages and abortions, IVF embryos, *ex profeso* embryos, cultures, cadavers, tissues and somatic cells. Care and preservation of progenitor cells. Patents

Applications	Basic research, characterization, biological behavior, differentiation, migration, neurodevelopment. Transplants, diseases of neurodevelopment, trauma, stroke, neurodegenerative diseases. Regenerative therapy. Risks, long term results. Public health impact. Patents and accessibility
